# Minimal Hepatic Encephalopathy and Biejia-Ruangan Are Associated with First Hospital Readmission in Nonalcoholic Cirrhosis Patients

**DOI:** 10.1155/2021/6652858

**Published:** 2021-05-07

**Authors:** Ting-Ting Jiang, Xiao-Li Liu, Yu-Yong Jiang, Xian-Bo Wang, Zhi-Yun Yang

**Affiliations:** Center of Integrative Medicine, Beijing Ditan Hospital, Capital Medical University, Beijing 100015, China

## Abstract

*Introductionand Aim*. Patients with cirrhosis are often hospitalized repeatedly for a variety of complications. This retrospective study aimed to assess the effects of minimal hepatic encephalopathy (MHE) and Biejia-Ruangan (BR) on first hospital readmission in nonalcoholic cirrhosis patients without previous overt hepatic encephalopathy (OHE) or hepatocellular carcinoma (HCC). *Materials and Methods*. A total of 176 hospitalized patients with nonalcoholic cirrhosis were included in this retrospective study. Patients who were first admitted to Beijing Ditan Hospital of Capital Medical University from January 2017 to September 2019 were enrolled. The primary endpoint was their first liver-related hospital readmission. The risk factors for readmission were analyzed by Cox proportional hazard regression analysis. *Results*. A total of 176 nonalcoholic cirrhosis patients without previous OHE or HCC were included; 57 patients (32.4%) were diagnosed with MHE, and 63 patients (35.8%) were administered BR (2 g, three times a day). Multivariate analysis revealed that nonalcoholic cirrhosis patients with MHE (HR, 5.805; 95% CI, 3.007–11.206; *x*, *P* < 0.001) and a higher Model for End-Stage Liver Disease (MELD) score (HR, 1.145; 95% CI, 1.068–1.227; *P* < 0.001) had an increased risk of first hospital readmission, and patients treated with BR (HR, 0.318; 95% CI, 0.151–0.670; *P*=0.003) had a decreased risk of first hospital readmission. *Conclusion*. MHE increased the risk of hospital readmission in nonalcoholic cirrhosis patients without previous OHE or HCC, and this risk was decreased by BR administration.

## 1. Introduction

Patients with cirrhosis are often readmitted to the hospital because of various complications. One of the most serious complications is hepatic encephalopathy (HE). Minimal hepatic encephalopathy (MHE), which is the mildest form on the hepatic encephalopathy spectrum, is usually not easily detected. There are no typical neurological and psychiatric manifestations in MHE patients, and MHE can only be detected through neuropsychological or neurophysiological tests [1]. Patients with MHE appear normal, while there are hidden dangers associated with driving and falling that affect their quality of life (QOL) [2–5]. Worldwide, the prevalence of MHE ranges from 30% to 84% in patients with cirrhosis [6–8]. Neurophysiological tests take a substantial amount of time, and clinicians are often too busy to screen patients with cirrhosis [9, 10]. In recent years, some studies have shown that the clinical prognosis of MHE patients is worse than that of cirrhosis patients with normal neuropsychological test results, and the mortality of MHE patients is significantly elevated due to hepatocellular carcinoma (HCC) and liver-related complications [11–13]. During clinical follow-up, a large proportion of MHE patients develop overt hepatic encephalopathy (OHE) and other complications. Once severe hepatic encephalopathy occurs, the one-year survival rate is less than 50%, and the three-year survival rate is less than 25% [14].

Some studies have revealed that smoking, the Child–Pugh score (CTP), the Model for End-Stage Liver Disease (MELD) score, the serum albumin (ALB) level, and the ammonia level are associated with the presence of MHE [11, 15, 16]. Other studies reported that age, gender, albumin level, HCC stage, MHE, CTP, and MELD scores were related to mortality [17, 18]. However, those studies always involved patients with alcoholic cirrhosis, previous OHE, and/or HCC, which may have affected the clinical outcomes [19–21]. Therefore, we rigorously excluded patients with alcoholic cirrhosis, previous OHE, or HCC in our study.

Traditional Chinese medicine (TCM), as an ancient medical system, has been applied worldwide [22]. Clinical trials have demonstrated that BR can block the development of hepatic fibrosis and reverse early cirrhosis [23, 24]. According to TCM theory, the pathogenesis of cirrhosis is weakened qi and blood, accompanied by blood stasis [25]. Biejia-Ruangan (BR), which is composed of at least 32 antifibrotic compounds, was formulated to promote qi and blood and invigorate the blood circulation to remove blood stasis [26]. The China Food and Drug Administration (CFDA) approved BR (Inner Mongolia Furui Medical Science Co Ltd, Wulanchabu, China, license number Z1999101) as the first TCM antifibrotic regimen to treat cirrhosis caused by chronic hepatitis B (CHB).

This study was carried out to determine the risk factors for the diagnosis and prognosis of MHE in nonalcoholic cirrhosis patients without previous OHE or HCC and to evaluate the effect of BR on the prognosis of these cirrhosis patients.

## 2. Materials and Methods

### 2.1. Study Design and Patients

In this retrospective study, we enrolled a total of 237 nonalcoholic cirrhosis patients who had been first admitted to our hospital between January 2017 and September 2019. Of these patients, 61 were excluded due to the following reasons: HCC (*N*=16), OHE or a history of OHE (*N*=3), drug use (*N*=14), fever (*N*=3), alcohol abuse (*N*=20), vision problems, or refusal to undergo the test (*N*=5). A total of 176 patients met the eligibility criteria. All patients meeting the criteria were included.

The inclusion criteria were as follows: hospitalized nonalcoholic cirrhosis patients between 20 and 75 years of age who underwent testing for MHE according to the psychometric hepatic encephalopathy score (PHES). The exclusion criteria were as follows: (1) the presence of OHE or a history of OHE; (2) a history of alcohol abuse; (3) the presence of HCC; (4) infection or spontaneous bacterial peritonitis (SBP) in the past 4 weeks; (5) gastrointestinal bleeding in the past 4 weeks; (6) the presence of neurological diseases; (7) the presence of psychiatric disorders or severe comorbidities; (8) use of drugs when the tests were performed, including benzodiazepines, antiepileptic or psychotropic substances, probiotics, rifaximin, and ammonia-lowering drugs (L-ornithine-L-aspartate, lactulose, and lactitol); (9) inability to undergo testing due to a lack of education or vision problems such as glaucoma, cataract, or other reasons; (10) a history of shunt surgery or the insertion of a transjugular intrahepatic portosystemic shunt; (11) use of TCM treatments other than BR; and (12) incomplete clinical data.

BR consists of more than 32 antifibrotic compounds [24]. The major ingredients of BR are shown in Table 1.

The definition of BR therapy (BR users) was as follows: medical records of BR use (a dosage of 2 g of BR three times a day for at least seven days during hospitalization and at least a total of six months of BR administration after discharge from the hospital).

  The definition of BR-6 therapy was as follows: a duration of BR therapy of six months
  The definition of BR-12 therapy was as follows: a duration of BR therapy of twelve months

### 2.2. Diagnosis of Minimal Hepatic Encephalopathy

All enrolled patients underwent PHES assessment, which consists of five subtests, namely, number connection tests (NCTs) A and B, a digit symbol test (DST), a line tracing test (LTT), and a serial dotting test (SDT). These tests were administered by well-trained specialists. Patients with liver cirrhosis were diagnosed with MHE if the results of more than two subtests were abnormal [27, 28].

### 2.3. Outcomes

The data were tracked via our electronic medical system with confirmation via telephone calls made by the researcher. The time from the initial inpatient screening to the first liver-related rehospitalization was recorded in months. Hospitalizations due to periodic evaluation were not included. Liver-related rehospitalizations were defined as those related to cirrhosis complications (hepatorenal syndrome, variceal bleeding, ascites and related spontaneous bacterial peritonitis, encephalopathy, and jaundice) and HCC. The primary endpoint was the first liver-related rehospitalization.

### 2.4. Clinical Data Collection

Cirrhosis was defined by ultrasound, CT scan, MRI, endoscopic analysis, biochemical parameters, and liver biopsy, if available. Liver dysfunction was evaluated by the MELD scores and the CTP. Blood samples were obtained from patients within 7 days of the MHE test. Demographic and laboratory parameters were collected for all patients from the electronic medical system, including gender, age, white blood cell (WBC) count, hemoglobin (HGB) level, thrombocyte (PLT) count, alanine transaminase (ALT) level, aspartate aminotransferase (AST) level, total bilirubin (TBIL) level, serum albumin (ALB) level, creatinine (Cr) level, international normalized ratio (INR), potassium (K) level, and sodium (Na) level. The etiology of cirrhosis was categorized into hepatitis B infection, hepatitis C infection, autoimmune-associated cirrhosis, and other cryptogenic cirrhosis. Compensated cirrhosis was defined as cirrhosis without complications. Decompensated cirrhosis was defined as cirrhosis with complications such as hepatorenal syndrome, variceal bleeding, ascites and related spontaneous bacterial peritonitis, encephalopathy, and jaundice.

All cirrhotic patients were treated according to the European Association for the Study of the Liver (EASL) clinical practice guidelines [29–32]. All procedures performed in studies involving human participants were in accordance with the ethical standards of the institutional and/or national research committee and with the 1975 Helsinki declaration and its later amendments or comparable ethical standards.

### 2.5. Statistical Analysis

Statistical analysis was performed using IBM SPSS version 23 (IBM Corp, NY) and Stata (Stata Corp LLC). Normally distributed continuous data were expressed as the mean ± standard deviation (SD) and were analyzed by Student's *t*-tests. Nonnormally distributed continuous data were presented as the median and interquartile range (IQR) and were analyzed using the Mann–Whitney U test. Categorical data were expressed as frequencies and were analyzed using the chi-square test and Fisher's exact test. The risk factors for MHE were analyzed by logistic regression analyses. The risk factors of first hospital readmission were analyzed by multivariate Cox regression analyses. Rehospitalization risk factors were analyzed by the Kaplan–Meier method and the log-rank test. Cut-offs for continuous variables were based on the Youden index. Two-sided *P* values <0.05 were considered statistically significant.

## 3. Results

### 3.1. Baseline Characteristics

In total, there were 176 consecutive patients, including 103 men and 73 women (age range, 24–73 years; 51.1 ± 10.7). The etiology of liver cirrhosis included hepatitis B virus infection (*N*=136), hepatitis C virus infection (*N*=14), autoimmune hepatitis (*n*=17), and cryptogenic cirrhosis (*N*=9). The numbers of cirrhotic patients in Child–Pugh classes A, B, and C were 126 (71.6%), 42 (23.9%), and 8 (4.5%), respectively. No significant difference was found between the BR group and no-BR group (*P* > 0.05) (Table 2).

### 3.2. Factors Associated with Minimal Hepatic Encephalopathy among the 176 Nonalcoholic Cirrhosis Patients

Among the 176 eligible patients, 57 (32.4%) were diagnosed with MHE. The prevalence rates of MHE in patients in Child–Pugh classes A, B, and C were 33 of 126 (26.2%), 18 of 42 (42.9%), and 6 of 8 (75%), respectively. In the univariate analysis, MHE was closely associated with serum albumin level, serum Na level, serum K level, and BR use. A logistic regression analysis was performed using age, ALT, AST, INR, MELD, serum Cr, ALB level, TBIL, serum Na level, serum K level, WBC count, and BR as variables. Only the ALB level was significantly associated with the presence of MHE (HR, 0.92; 95% CI, 0.852–0.993; *P*=0.032) (Table 3). A decreased ALB level was related to a greater risk of MHE in nonalcoholic cirrhosis patients.

### 3.3. Factors Associated with First Hospital Readmission among the 176 Nonalcoholic Cirrhosis Patients

Patients were followed for a median of 17 months (IQR, 5.3, 26.8). During the study period, 45 (25.6%) patients were first readmitted. In total, four patients died during their first readmission; two patients died of jaundice, one patient died of hepatorenal syndrome, and another patient died of ascites and related spontaneous peritonitis. The first hospital readmission events included 5 cases of encephalopathy, 5 cases of HCC, 17 cases of ascites and related spontaneous peritonitis, 14 cases of gastrointestinal bleeding, 3 cases of jaundice, and 1 case of hepatorenal syndrome.

**Figure 1:**
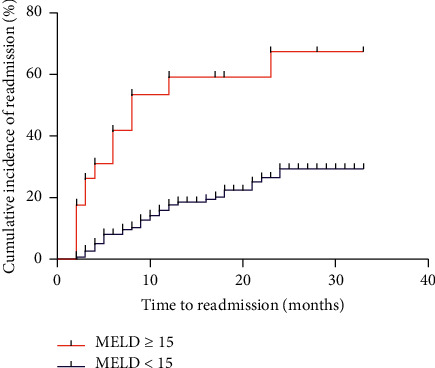
There was marked difference in first hospital readmission risk between the MELD ≥15 group and the MELD <15 group (*P* < 0.001) (*N*=176).

The prognostic factors in nonalcoholic cirrhotic patients are summarized in Table 4. Univariate Cox regression analysis revealed that MHE, BR use, the MELD score, serum Na level, ALB level, and TBIL level were risk factors for first hospital readmission. The multivariate analysis identified nonalcoholic cirrhotic patients with MHE (HR, 5.805; 95% CI, 3.007–11.206; *P* < 0.001) and a higher MELD score (HR, 1.145; 95% CI, 1.068–1.227; *P* < 0.001) as associated with a poor prognosis and patients treated with BR (HR, 0.318; 95% CI, 0.151–0.670; *P*=0.003) as having a favorable prognosis (Table 4). The cut-off value (specificity + sensitivity-1) for the MELD score was 15. There was marked difference in first hospital readmission risk between the MELD ≥15 group and the MELD <15 group (*P* < 0.001) (Figure 1).

### 3.4. MHE and First Hospital Readmission in the 176 Nonalcoholic Cirrhosis Patients

**Figure 2:**
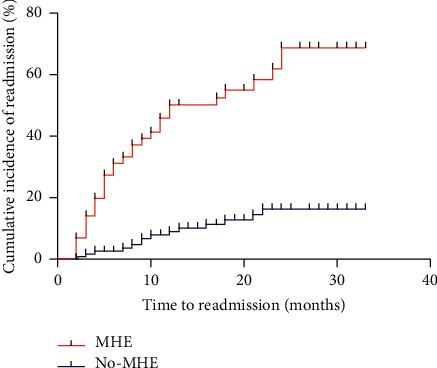
The cumulative incidence of first hospital readmission was significantly higher in the MHE group than that in the no-MHE group (*P* < 0.001) (*N*=176).

The first hospital readmission events in the MHE group included 4 cases of encephalopathy, 2 cases of HCC, 14 cases of ascites and related spontaneous peritonitis, 8 cases of gastrointestinal bleeding, 3 cases of jaundice, and 1 case of hepatorenal syndrome. In the no-MHE group, there were 3 cases of HCC, 3 cases of ascites and related spontaneous peritonitis, 6 cases of gastrointestinal bleeding, and 1 case of encephalopathy. When all first hospital readmission events during the follow-up were considered, there were significantly more events in the MHE group, with 32 events (0.56 events per patient) vs. 13 events in the no-MHE group (0.11 events per patient) (*P* < 0.001). The cumulative incidence of first hospital readmission was significantly higher in the MHE group than in the no-MHE group (*P* < 0.001) (Figure 2).

### 3.5. BR and First Hospital Readmission in the 176 Nonalcoholic Cirrhosis Patients

**Figure 3:**
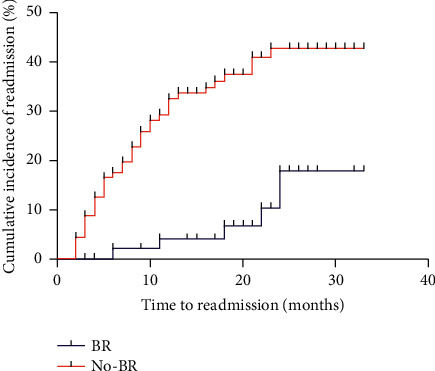
The nonalcoholic cirrhotic patients treated with BR were at a lower risk of first hospital readmission than those without BR treatment (*P*=0.004P=0.004) (*N*=176).

The first hospital readmission events in the BR group included 1 case of HCC, 3 cases of ascites and related spontaneous peritonitis, 3 cases of gastrointestinal bleeding, and 2 cases of encephalopathy. The first hospital readmission events in the no-BR group included 3 cases of encephalopathy, 4 cases of HCC, 14 cases of ascites and related spontaneous peritonitis, 11 cases of gastrointestinal bleeding, 3 cases of jaundice, and 1 case of hepatorenal syndrome. When all first hospital readmission events during follow-up were considered, there were significantly more events in the no-BR group, with 36 events (0.32 events per patient) vs. 9 events in the BR group (0.14 events per patient) (*P*=0.01). The cumulative incidence of first hospital readmission was significantly higher in the no-BR group than in the BR group (*P*=0.004) (Figure 3).

**Figure 4:**
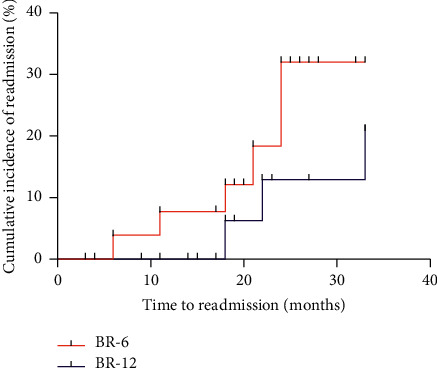
No significant difference was found in the cumulative incidence of first hospital readmission between the BR-6 and BR-12 groups (*P*=0.202) (*N*=63).

Of the 63 patients who were administered with BR, the duration was six months in 26 patients and twelve months in 37 patients. The first hospital readmission events in the BR-6 therapy group included 2 cases of encephalopathy, 1 case of HCC, 2 cases of ascites and related spontaneous peritonitis, and 1 case of gastrointestinal bleeding. The first hospital readmission events in the BR-12 therapy group included 1 case of ascites and related spontaneous peritonitis and 2 cases of gastrointestinal bleeding. No significant difference was found in the cumulative incidence of first hospital readmission between the BR-6 and BR-12 groups (*P*=0.202) (Figure 4).

## 4. Discussion

Our study led to three major conclusions. First, a decreased ALB level was related to a higher risk of the presence of MHE in nonalcoholic cirrhosis patients without previous OHE or HCC. Second, MHE and the MELD score were risk factors associated with first hospital readmission in nonalcoholic cirrhosis patients. Third, BR helped to decrease the risk of hospital readmission in nonalcoholic cirrhosis patients. No significant difference was observed in the cumulative incidence of first hospital readmission between the groups using BR for 6 months and 12 months.

In our study, the prevalence of MHE in nonalcoholic cirrhosis patients was 32.4%, which is lower than that in the general population [8, 28]. The reason lies in that patients with alcohol-related liver disease were excluded from our study, and alcohol abuse may be a risk factor that affects the results of neuropsychological tests. The effect of alcohol on cognitive function is clear; however, the psychometric alterations in liver cirrhosis patients with MHE due to alcohol abuse are still controversial [7, 33–38]. The development of HCC may accelerate the course of the disease at any stage [21], and patients with previous OHE often have worse cognitive function [7, 39, 40]. Therefore, this study excluded patients with alcohol-related liver cirrhosis and previous OHE and HCC to evaluate the prognosis of patients with liver cirrhosis more precisely.

Among the variables analyzed in this study, the multivariate analysis demonstrated that a decreased ALB level was independently associated with the presence of MHE in nonalcoholic cirrhosis patients. Some studies have concluded that a decreased ALB level may be associated with an increased risk of OHE during hospitalization in cirrhosis patients [41, 42]. Other studies have reported that the ALB level is an independent risk factor associated with the development of covert hepatic encephalopathy (CHE) [43–45]. In contrast to these studies, our study excluded patients with alcoholic cirrhosis and previous OHE, which may affect the diagnosis of MHE. Moreover, the Child–Pugh classification, which consists of many clinically significant variables in addition to ALB, was also excluded in our multivariate analysis.

MHE is associated with severe liver-related problems and increased mortality. Ampuero et al. [17] suggested that MHE was associated with a reduced 5-year survival rate in patients with liver cirrhosis. Hanai et al. [11] indicated that MHE was associated with an increased risk of mortality in patients with liver cirrhosis, independent of HCC stage or CTP by a propensity score-matching analysis. Barone et al. [46] revealed that the critical flicker frequency (CFF), which is used to diagnose MHE, can be used to predict the mortality risk. Our research concluded that MHE was the most important factor associated with the first hospital readmission of nonalcoholic cirrhosis patients. In our study, we strictly excluded patients with previous OHE and HCC, which may affect the clinical outcome. We drew a conclusion that MHE was closely associated with first hospital readmission in nonalcoholic cirrhosis patients.

The MELD score has been widely applied to assess the severity and prognosis of liver disease. Fung et al. [47] concluded that the MELD score at any time point can accurately predict short-term mortality in patients with severe flares of CHB. Volk et al. [48] found that the predictors of time to first readmission among patients with decompensated cirrhosis included the MELD score, serum Na level, and number of medications at discharge. D'Amico et al. [21] reported that the MELD score was a predictor of long-term survival in patients with decompensated cirrhosis. In our study, the MELD score was also demonstrated to be risk factor associated with the first hospital readmission in nonalcoholic cirrhosis patients.

In recent decades, the role of TCM in the treatment and prevention of fibrosis has been confirmed by a growing number of experiments and clinical studies [25, 49, 50]. BR, which was the first antifibrotic herb approved by the CFDA to treat fibrotic liver disease in China, has been applied for clinical treatment for many years. BR exerts its antifibrotic effect via several mechanisms [51–53]. Recently, a multicenter, randomized, double-blind, placebo-controlled trial showed that addition of BR to nucleoside analog (NAs) in CHB patients with advanced fibrosis or cirrhosis can improve liver fibrosis regression [54]. Our study found that BR delayed the occurrence of complications of cirrhosis and improved the QOL of nonalcoholic cirrhosis patients without previous OHE or HCC. However, we did not find a significant difference in the cumulative incidence of first hospital readmission between the groups using BR for 6 months and 12 months. Recently, no research has showed the relationship between BR and MHE. However, a recent report has reported that TCM (Babao Dan) can improve neurocognitive function by targeting and regulating TLR4 inflammatory pathway in MHE patients [55]; a further study about the effect of BR on MHE may be explored.

This study also has several limitations. First, the study was a retrospective cohort study; patients with serious complications are often too weak to take pills, which may lead to bias in clinical characteristics between BR users and non-BR users. Second, this was a single-center study, and the number of patients involved in this study was limited. We expect that multicenter and large-sample randomized controlled trials (RCTs) will be conducted in the future.

In conclusion, MHE is associated with a poor prognosis in nonalcoholic cirrhosis patients. It is essential for doctors to screen for MHE in liver cirrhosis patients using neuropsychological/neurophysiological tests. MHE patients often have an elevated risk of hospital readmission; therefore, it is very important for clinicians to treat MHE patients in a timely manner. BR can decrease the risk of hospital readmission in nonalcoholic cirrhosis patients without previous OHE or HCC. BR therapy may play an important role in improving the prognosis of cirrhosis patients, but this finding still needs to be verified by prospective RCTs in the future.

## Figures and Tables

**Table 1:** The major ingredients of Biejia-Ruangan (BR).

Chinese phonetic alphabet name	English name
Bejia	Turtle shell
Chishao	Peony root
Ezhu	Zedoray rhizome
Danggui	*Angelica sinensis*
Dangshen	*Campanumaea pilosula*
Huangqi	*Astragalus*
Sanqi	Pseudo-ginseng
Dongchongxiacao	Plant worms
Ziheche	Dried human placenta
Banlangen	*Baphicacanthus* root
Lianqiao	Farsythio

**Table 2:** Clinical characteristics of patients with and without Biejia-Ruangan (BR) treatment in nonalcoholic cirrhosis.

	All patients (*N*=176)		
	BR (*N*=63)	No-BR (*N*=113)	*P* value
Age, mean ± SD (range) (yr)	50.8 ± 11.1	52.3 ± 10.3	0.745
Gender (m/f)	37/26	66/47	0.967
*Etiology of cirrhosis*			0.794
Hepatitis B	49	87	
Hepatitis C	6	8	
Autoimmune hepatitis	6	11	
Unknown	2	7	
Serum albumin (g/L)	35.5 ± 5.1	35.8 ± 5.8	0.090
Decompensated cirrhosis	45/18	87/26	0.414
K + (mmol/L)	3.82 ± 0.39	3.95 ± 0.37	0.133
*Child–Pugh class*			0.967
A	45	81	
B	15	27	
C	3	5	
ALT (U/L)	55.5 (29.2, 21.9)	42.5 (23.5, 17.2)	0.069
AST (U/L)	54.5 (36.9, 39.9)	58.4 (28.8, 18.9)	0.084
Total bilirubin (mg/dL)	32.4 (21.5, 21.6)	33.8 (19.5, 14.3)	0.408
Na + (mmol/L)	140.7 ± 2.9	140.0 ± 3.6	0.716
INR	1.26 (1.23, 0.25)	1.30 (1.21, 0.33)	0.982
Creatinine (*μ*mol/L)	64.7 (57.2, 20.5)	64.9 (63.6, 17.2)	0.130
Hemoglobin (g/L)	121.9 ± 24.9	113.1 ± 23.2	0.396
Platelet (10^9^/L)	92.8 (73.3, 76)	85.6 (60.0, 63.0)	0.526
White blood cells (10^9^/L)	3.53 (3.15, 2.55)	3.53 (3.28, 1.84)	0.704
MELD	10.9 (10.0, 5.0)	10.6 (9, 4)	0.389

Abbreviations: K+, potassium; Na+, sodium; ALT, alanine aminotransferase; AST, aspartate aminotransferase; INR, international normalized ratio; MELD, model for end-stage liver disease; BR, Biejia-Ruangan.

**Table 3:** Univariate and multivariate regression analysis for predictors of minimal hepatic encephalopathy (MHE) in nonalcoholic cirrhosis patients (*N* = 176).

Variable	OR	95% variable OR CI for EXP (B)		*P* value	OR	95% variable OR CI for EXP (B)		*P* value
		Lower	Upper			Lower	Upper	
Age (year)	1.109	0.989	1.050	0.214	1.031	0.995	1.069	0.095
ALT (U/L)	1	0.997	1.003	0.941	0.987	0.965	1.010	0.260
AST	1.001	0.998	1.003	0.475	1.010	0.994	1.027	0.205
Na + (mmol/L)	0.893	0.802	0.994	0.039	0.924	0.815	1.047	0.215
K + (mmol/L)	2.744	1.121	6.716	0.027	2.496	0.923	6.749	0.072
Serum albumin (g/L)	0.925	0.872	0.982	0.010	0.920	0.852	0.993	0.032
Total bilirubin (*μ*mol/L)	1.006	0.998	1.015	0.130	1.004	0.990	1.018	0.592
INR	0.511	0.139	1.874	0.311	0.580	0.141	2.383	0.450
Creatinine (*μ*mol/L)	1.002	0.989	1.015	0.783	1.002	0.986	1.019	0.801
White blood cells (10^9^/L)	0.943	0.774	1.15	0.563	0.926	0.735	1.166	0.514
BR	0.465	0.23	0.941	0.033	0.479	0.223	1.028	0.059
MELD	1.074	0.988	1.167	0.092	0.962	0.818	1.130	0.634

OR, odds ratio; CI, confidence interval; ALT, alanine aminotransferase; AST, aspartate aminotransferase; Na+, sodium; K+, potassium; INR, international normalized ratio; BR, Biejia-Ruangan; MELD, model for end-stage liver disease.

**Table 4:** Univariate and multivariate analyses to predict first hospital readmission in nonalcoholic cirrhosis patients (*N* = 176).

Variable	HR	95% variable HR CI for EXP (B)		*P* value	HR	95% variable HR CI for EXP (B)		*P* value
		Lower	Upper			Lower	Upper	
Age (year)	1.022	0.994	1.051	0.132	1.031	0.996	1.067	0.088
ALT	0.998	0.991	1.006	0.689	0.989	0.970	1.009	0.287
AST	1.001	0.998	1.003	0.649	1.008	0.994	1.022	0.276
Na	0.911	0.842	0.986	0.021	0.963	0.882	1.051	0.395
K	2.006	0.907	4.436	0.085	1.603	0.603	4.528	0.344
MHE	6.515	3.431	12.371	0	5.805	3.007	11.206	0
Serum albumin (g/L)	0.905	0.856	0.957	0	0.948	0.885	1.014	0.122
Total bilirubin (*μ*mol/L)	1.009	1.005	1.013	0	1.001	0.994	1.008	0.735
INR	0.815	0.263	2.523	0.723	1.251	0.349	4.490	0.731
Creatinine (*μ*mol/L)	1.004	0.994	1.015	0.406	0.996	0.981	1.010	0.547
White blood cells (10^9^/L)	0.937	0.780	1.126	0.487	0.997	0.803	1.239	0.982
BR	0.361	0.174	0.750	0.006	0.318	0.151	0.670	0.003
MELD	1.154	1.079	1.235	0	1.145	1.068	1.227	0

HR, hazard ratio; CI, confidence interval; ALT, alanine aminotransferase; AST, aspartate aminotransferase; Na+, sodium; K+, potassium; MHE, minimal hepatic encephalopathy; INR, international normalized ratio; MELD, model for end-stage liver disease; BR, Biejia-Ruangan.

## Data Availability

The data used to support the findings of this study are included within the article.
